# Dual-Seq reveals genome and transcriptome of *Caedibacter taeniospiralis*, obligate endosymbiont of *Paramecium*

**DOI:** 10.1038/s41598-020-65894-1

**Published:** 2020-06-16

**Authors:** Marcello Pirritano, Nestor Zaburannyi, Katrin Grosser, Gilles Gasparoni, Rolf Müller, Martin Simon, Martina Schrallhammer

**Affiliations:** 1 Molecular Cell Biology and Microbiology, University of Wuppertal, Wuppertal, Germany; 2 Molecular Cell Dynamics Saarland University, Saarbrücken, Germany; 3 Department of Microbial Natural Products, Helmholtz Centre for Infection Research and Department of Pharmacy, Helmholtz Institute for Pharmaceutical Research Saarland (HIPS), Saarland University, Saarbrücken and German Centre for Infection Research (DZIF), Hannover, Germany; 4 Microbiology, Institute of Biology II, Albert Ludwig University of Freiburg, Freiburg, Germany; 5 Genetics, Centre for Human and Molecular Biology, Saarland University, Saarbruecken, Germany; 6 Present Address: Deep Sequencing Unit, Max-Planck-Institute for Immunobiology and Epigenetics, Freiburg, Germany

**Keywords:** Coevolution, Molecular evolution, Phylogenetics, DNA, RNA, Bacterial evolution, Bacterial genetics, Bacterial genomics, Bacterial host response, Metagenomics, Symbiosis

## Abstract

Interest in host-symbiont interactions is continuously increasing, not only due to the growing recognition of the importance of microbiomes. Starting with the detection and description of novel symbionts, attention moves to the molecular consequences and innovations of symbioses. However, molecular analysis requires genomic data which is difficult to obtain from obligate intracellular and uncultivated bacteria. We report the identification of the *Caedibacter* genome, an obligate symbiont of the ciliate *Paramecium*. The infection does not only confer the host with the ability to kill other cells but also renders them immune against this effect. We obtained the *C. taeniospiralis* genome and transcriptome by dual-Seq of DNA and RNA from infected paramecia. Comparison of codon usage and expression level indicates that genes necessary for a specific trait of this symbiosis, i.e. the delivery of an unknown toxin, result from horizontal gene transfer hinting to the relevance of DNA transfer for acquiring new characters. Prediction of secreted proteins of *Caedibacter* as major agents of contact with the host implies, next to several toxin candidates, a rather uncharacterized secretome which appears to be highly adapted to this symbiosis. Our data provides new insights into the molecular establishment and evolution of this obligate symbiosis and for the pathway characterization of toxicity and immunity.

## Introduction

Symbionts can have severe impact on host nutrition, metabolism, reproduction, immune and stress responses, and even behavior. Our knowledge about their biology is mostly limited to pathogenic representatives of medical or economic concern. Another bias is the focus on the minority which can be cultivated in artificial media, most probably due to the technical difficulties when facing uncultivated prokaryotes. But these symbionts represent only a small proportion of the natural diversity. A fundamental resource for understanding the biology of uncultivated intracellular symbionts is their genome sequence. It can be used to infer key aspects such as metabolic properties, phylogeny and evolution as well as additional traits crucial for the symbiosis. Next generation sequencing (NGS) techniques now allow for obtaining genome sequences of uncultivated symbionts of less well-studied host organisms. Still, the procedure entails multiple challenges such as low DNA quality and quantities of mixed samples with unfavorable abundances of target DNA, unavailability of reference genomes, etc. Choosing the ciliate *Paramecium tetraurelia* carrying a cytoplasmic infection with *Caedibacter taeniospiralis* (Gammaproteobacteria), we provide a roadmap for sequencing the genome and transcriptome of obligate intracellular bacteria.

We focus on this system as *Paramecium* can host diverse bacterial endosymbionts and only few genomes are available so far (i.e. four*Holospora* draft genomes^1,2^ and the only ones completed, “*Candidatus* Fokinia solitaria”^3^ and “*Candidatus* Deianiraea vastatrix”^4^). All belong to the sister families *Rickettsiales* and *Holosporales*, thus *C. taeniospiralis* represents a member of another clade of Proteobacteria. So far, it is the only described gammaproteobacterial symbiont of *Paramecium*. It can serve as a phylogenetic distant example for genome adaptation during the transition from a free-living to an intracellular lifestyle as well as co-evolution with the same host and ecological restraints as the alphaproteobacterial symbionts. The interaction between *Paramecium tetraurelia* and *Caedibacter* is especially intriguing as it is linked to the killer trait^5,6^ which is the ability to eliminate symbiont-free paramecia^7^ gained from symbiosis with *Caedibacter* or *Caedimonas*^8^ bacteria. This trait is expressed by the bacterial symbionts. It comprises three components: the R-body, which constitutes an unusual protein delivery machine, an unidentified toxin, and an unknown resistance mechanism. If symbiont-free paramecia ingest released *Caedibacter*, the R-body is triggered to unroll and therewith delivers the toxin into the *Paramecium* cytoplasm. R-bodies themselves are not toxic and once the symbiont is eliminated the paramecia loose the resistance^9,10^. In addition, this symbiosis can result in increased host cell densities without the provision of nutritional supplements^10^. The mechanisms how the symbionts cause these host phenotype modifications are not yet fully understood. With the *C. taeniospiralis* genome annotation we provide here a RNA-Seq corrected annotation of the former draft genome sequence^11^ which was not sufficient for a solid annotation of genes and operons. Furthermore, we close several gaps by using additionally long reads from Oxford Nanopore Technologies (ONT). As a result, we are able to present a roadmap for assessing genomes of uncultivated symbionts, the first genome of a *Paramecium* symbiont outside Alphaproteobacteria, and a resource to address the question how this symbiont drastically changes the properties of its host.

## Results and Discussion

### Dual-Seq reveals the *Caedibacter* genome sequence and facilitates refined phylogenetic placement

**Figure 1:**
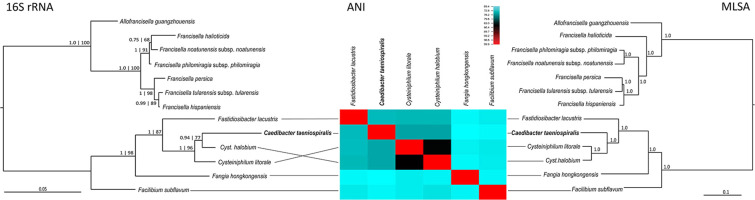
Phylogenetic analysis of *Caedibacter taeniospiralis*. Using the newly available *Caedibacter* genome (highlighted in bold), evolutionary reconstructions were carried out performing three different approaches of phylogenetic comparison based on 16S rRNA gene sequences (left), ANI analysis (middle) and MLSA comparison (right) considering close relatives of *Caedibacter*.

As *Caedibacter* cannot be cultivated outside of its host cell, we used total DNA isolated from food bacteria depleted paramecia cultures for library preparation using Tagmentation^11^. After assembly, we separated *Caedibacter* sequences from host chromosomes by GC percentage and coverage (Supplementary Fig. S1). The resulting assembly was further improved by ONT sequencing of high molecular weight DNA which allowed to close several gaps (Table 1). The resulting assembly consists of 17 scaffolds and one circular plasmid with a total size of 1.32 Mbp. Although we were not able to close the chromosome, this genome currently represents the best assembled genome (Table 2) inside a group of related bacteria (*Fastidiosibacteriaceae*^12^), now allowing for a more detailed phylogenetic characterization. We performed phylogenetic reconstructions (Fig. 1) based on 16S rRNA gene sequences and a concatenated alignment of 19 conserved protein-coding genes as well as genome-by-genome comparisons using average nucleotide identity (ANI) and digital DNA-DNA hybridization (Supplementary Table S1). The different analyses are in good agreement and place *C. taeniospiralis* within the little characterized family *Fastidiosibacteriaceae*, sister family to the facultative intracellular *Francisellaceae* which cause zoonotic diseases in fish and mammals. Notably, *Caedibacter* is the only organism within *Fastidiosibacteriaceae* with an obligate intracellular lifestyle, all other members were isolated from marine or freshwater samples and grow on artificial media. The closest relatives of *C. taeniospiralis* are *Cysteiniphilum litorale*^13^ and *Cysteiniphilum halobium*^14^.

**Table 1:** Properties of the assembled *Caedibacter taeniospiralis* genome prior to (first draft) and after annotation improvement by RNA-Seq and Oxford Nanopore sequencing (second draft).

	First Draft	Second Draft
Size	1.3 Mbp	1.32 Mbp
Contigs	24	18
GC-Content	41.5%	41.3%
Protein coding sequences	1080	1092
Functionally annotated proteins	787 (72%)	806 (73.8)
rRNA gene cluster	3	3
tRNA genes	36	36
Operons	not determined	202

**Table 2:** Comparison of *Fastidiosibacteraceae* with available genome sequences.

Organism	Size	Contigs	GC-Content	Protein coding sequences	rRNA gene cluster	tRNA
*Caedibacter taeniospiralis*	1.32 Mbp	18	41.3%	1092	3	36
*Cysteiniphilum litorale*	3.24 Mbp	198	38.4%	2850	8*	43
*Cysteiniphilum halobium*	2.44 Mbp	159	37.6%	2106	5*	41
*Fangia hongkongensis*	2.95 Mbp	38	37.9%	2655	5	38
*Fastidiosibacter lacustris*	2.07 Mbp	62	36.3%	1848	6*	38
*Facilibium subflavum*	2.87 Mbp	389	37.8%	2512	2*	39

rRNA cluster indicated with an * are partially incomplete or potentially split. Genome accession numbers are indicated in Supplementary Table S1.

### RNA-Seq corrected gene annotation

**Figure 2:**
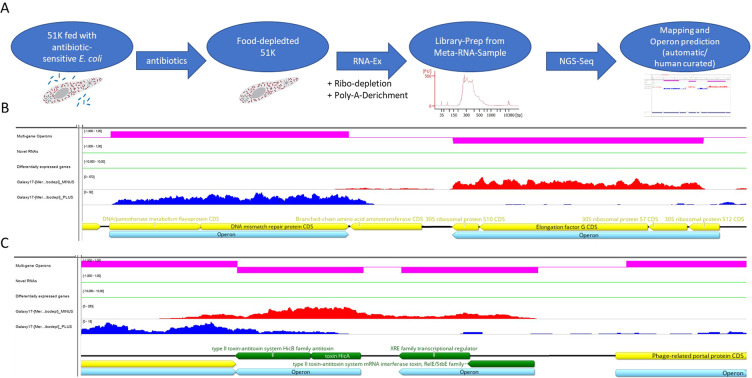
Improving gene annotation. (**A**) Workflow of operon prediction and annotation correction using mRNA-Seq data from meta-RNA-samples. (**B**,**C**) IGV snapshot of mapped meta-transcriptome reads to the *Caedibacter taeniospiralis* genome. Shown are (from top to bottom) the automatically predicted operon structures (pink bars, direction indicated by position above or below the pink line), automatically predicted novel RNAs and differentially expressed genes (in green) and antisense (red) and sense (blue) directed read coverage. Genes previously annotated are colored in yellow, green annotated genes were not annotated prior to annotation improvement, but were found manually due to mRNA coverage. Predicted operons are annotated in light blue.

Gene annotation was carried out by analysing the genome assembly which revealed 1080 protein coding gene candidates, 36 tRNAs and 3 rDNA cluster^11^ (Table 1). Dual RNA-Seq was carried out with the aim to improve this annotation and to verify transcriptional units and operons. Thus, prokaryotic mRNA was enriched for by food depletion using antibiotics as described previously^11^ as well as subsequent double depletion of host and symbiont rRNA and host poly(A)RNA (Fig. 2A). We created directional RNA libraries to dissect which strand of the genome is transcribed. The Rockhopper tool^15^ was applied to define transcriptional units and operons based on the obtained RNA-Seq information. Accordingly, we improved the annotation by correcting individual ORFs and increased the number of protein coding genes to 1.091 and predicted 202 operons. Furthermore, we distinguished gene annotations of automatically annotated genes and human curated ones (see Fig. 2B,C). The latter involved manual inspection of the predicted protein sequences via database comparison (NCBI nucleotide collection respectively non-redundant protein sequences) as well as *de novo* annotation of previously not predicted protein sequences based on mRNA signals. Figure 2B,C show examples for annotation improvements in the Integrated Genomics Viewer (IGV) browser^48^.

**Figure 3:**
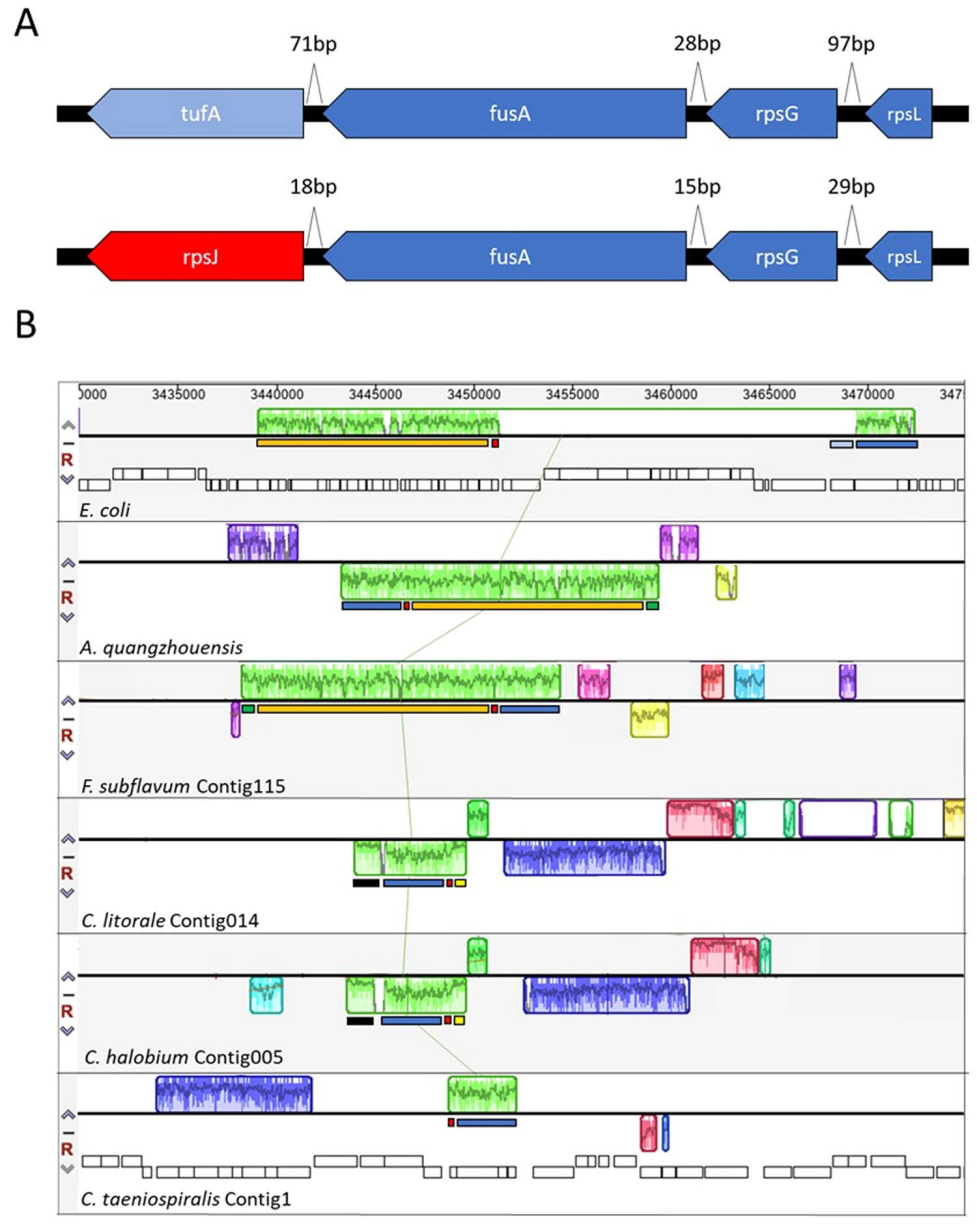
(**A**) *E. coli str*-operon (top) and a homolog operon in *Caedibacter taeniospiralis* (bottom). Genes that are in common between the two operons (ribosomal protein *rpsL* and *rpsG* as well as elongation factor *fusA*) are labeled in dark blue, varying genes are colored differently. Length of intergenetic spaces are indicated in basepairs. (**B**) Synteny of the *str*-operon and its genetic context between different relatives of *C. taeniospiralis* and *E. coli*. Homologous loci between species are indicated by colored squares. The presence of certain sequences in different species are indicated by a coverage blot within those squares, a lack of those by a blank space. The light green square (centered) represents the genomic context of the *str*-operon. To facilitate the comparison of the synteny of the genetic context, the presence and absence of homologous sequences within the different species were labeled with different colored bars.

In addition to gene content, also genome synteny and operon composition now enable the comparison of the symbiont genome to free-living species and thus provide insights into the evolutionary adaption of the killer trait symbiosis. Figure 3A shows the comparison of the *C. taeniospiralis str*-operon juxtaposed to *E. coli*^16^ indicating the exchange of the elongation factor EF-G (*tufA*) against the ribosomal protein S10 (*rpsJ*) in *Caedibacter*. The replacement of the elongation factor which is encoded on a different contig (CDBSP s08) may have led to a distorted expression level as consequence of the destroyed co-transcription. When comparing the respective operon structure in the sequenced *Fastidiosibacteriaceae* and *Caedibacter*, an interesting evolution of its synteny can be observed (Fig. 3B). The *str*-operon composition of *Caedibacter* is a general feature of the *Fastidiosibacteriaceae*. Thus, this composition and obvious difference to *E. coli* has clearly not evolved as result of the symbiotic adaption of *Caedibacter*. Considering the organization of *E. coli* as ancestral, the shift towards the replacement of *tufA* with *rpsJ*, and general reorganization as present in *Caedibacter* potentially correlates with an increasing fastidiousness^12,13^ of the bacteria regarding their cultivation or growth conditions. The rearrangement of the *str*-operon might be interpreted as one example for a decrease in regulatory fine-tuning in *Fastidiosibacteriaceae* which results in the strict dependence on the rather constant environment of a host organism such as *Paramecium* cytoplasm in case of *Caedibacter*. For this symbiont it was shown that even small changes of the cultivation temperature of the “poikilothermic” ciliates can have drastic effects to the symbiont density^17^.

### Horizontal gene transfer as driver of intracellularity

While the majority of *Paramecium* endosymbionts belongs to a fast evolving branch of obligate intracellular Alphaproteobacteria, *Caedibacter’s* closest relatives are free-living. Typical hallmarks for the transition from free-living to intracellular life such as reduced genome size, gene loss, and fewer rRNA genes^18^ are evident in the genome of *Caedibacter* (Table 2). Interestingly, the typical positive correlation between a reduction in genome size and GC-content^18,19^ is not detectable for *Fastidiosibacteriaceae*, as it is highest for the smallest genome (Table 2). While the importance of horizontal gene transfer (HGT) for rapid adaption and evolution of prokaryotes is broadly accepted, it has been suggested that obligate endosymbiotic bacteria might be protected from mobile genetic elements and DNA exchange due to their intracellular lifestyle. But as several studies demonstrate, e.g.^20–22^, evidence for different kinds of HGT can be detected in endosymbionts. Thus, we searched the genome of *Caedibacter* for horizontally acquired genes involved in its symbiotic interaction with *Paramecium*. A fascinating question remains unanswered: Were these genes acquired prior to the transition to an intracellular lifestyle and actually enabled the free-living ancestor of *Caedibacter* to become an endosymbiont? Or did the HGT occur when *Caedibacter* already lived inside *Paramecium* and hence stabilized the symbiosis by e.g. the acquisition of the killer trait? Double infections with different symbionts have been reported for *Paramecium*, so this remains an intriguing hypothesis^8,23,24^.

It has been speculated that central elements of the here described symbiosis, i.e. the Reb genes encoding for the R-body, have been acquired via HGT^6,8,25^. The R-body protein delivery machinery is produced by the obligate *Paramecium* endosymbionts *Caedibacter*, belonging to Gammaproteobacteria and *Caedimonas*, member of Alphaproteobacteria^8^. In the latter, phage-like particles are often found associated to the R-body structure by transmission electron microscopy^5^. Thus, we searched the genome of *Caedibacter* for the presence of mobile genetic elements and other indications for HGT, which might also assist in the search for and identification of the toxin and resistance mechanism of the killer trait.

**Figure 4:**
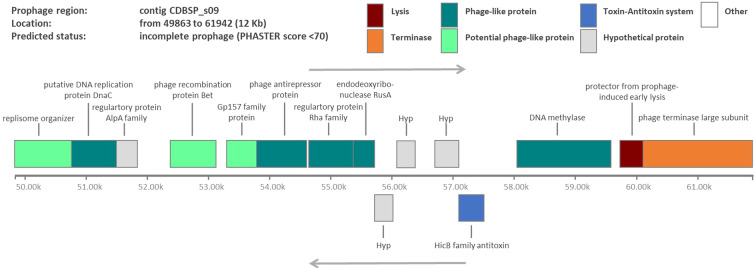
Identified incomplete prophage in the *C. taeniospiralis* genome. This incomplete prophage of ca. 12 kbp is localized on contig CDBSP s09 and consists of several phage-like proteins (bright and dark green), a protein involved in phage-induced lysis (red), a terminase (orange) as well as the HicB antitoxin (blue).

We confirmed the presence of a circular plasmid (pKAP51, 41.65 kb) that carries the Reb operon encoding for the R-body. This plasmid shares high similarities to plasmid pKAP298 (49.11 kb) of another strain of *C. taeniospiralis*, strain 298^26^. It encodes hypothetical proteins, transposases and phage-derived genes. The main difference to plasmid pKAP298 is the excision of transposon Tn5403 (7.78 kb) which is lacking from pKAP51. As phages have been implicated to play a role in the killer trait either by structural evidence^5,6^ or indirect experimental proof such as increase R-body production after UV-irradiation or induction with mitomycin C^27^, we searched for phage genomes on the bacterial chromosome. We applied PHASTER (PHAge Search Tool Enhanced Release^28^), to identify prophage sequences within the *Caedibacter* genome and detected one incomplete prophage of ca. 12 kb (Fig. 4). Noteworthy, this potential prophage encodes a component of a toxin-antitoxin system, HicB. Interestingly, *C. taeniospiralis* also possesses phage defense mechanisms as we identified a CRISPR locus headed by a AT-rich leader sequence which serves as promoter for the pre-crRNA synthesis followed by three identical repeats and 2 unique sequences, the so-called spacers (Supplementary Fig. S2). Together with a set of 7 CAS (CRISPR-associated) Type IC proteins, they constitute a CRISPR immune system. CRISPR are specific structures found in the majority of archaeal and many bacterial genomes that show characteristics of both tandem and interspaced repeats.

**Figure 5:**
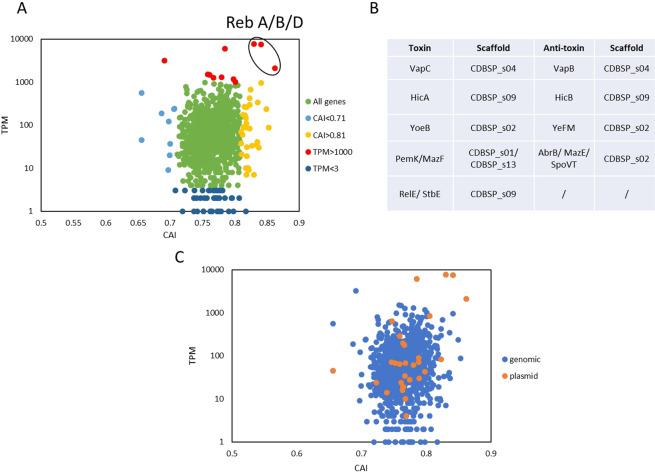
Genes with potential relevance for the *Caedibacter-Paramecium* symbiosis. (**A**) Codon adaptation (CAI) vs. gene expression (transcripts per million, TPM) values are compared. Genes with a particular high or low CAI (greater than 0.81 in yellow, below 0.71 in light blue) or TPM value (greater than 1000 in red, below 3 in dark blue) are highlighted accordingly. The Reb genes RebA, RebB and RebD are encircled. (**B**) List of annotated toxin-antitoxin systems present in the *Caedibacter* genome. (**C**) Dissection of gene expression between genomic and plasmid genes in relation to CAI and gene expression.

As mentioned before, an unknown toxin, which is delivered by the R-body, kills uninfected paramecia and hence is central to the symbiosis between *Caedibacter* and *Paramecium*. Additionally, the symbiosis provides immunity against this toxin raising the question for the underlying mechanism. This toxin shows a surprising high specificity for members of the genus *Paramecium*^7^, possibly correlating with the host specificity of the toxin producing bacteria^9^. Our genome annotation enables reverse genetics for the unknown toxin to understand the specificity to individual cells and furthermore the identification of the conditional resistance mechanism, as the immunity towards the killer trait is lost when the symbionts are eliminated from the killer paramecia. We started this analysis by plotting the codon adaptation index (CAI) of protein coding genes against gene expression. Figure 5A shows that there are indeed discrete outliers regarding the CAI, especially the highlighted Reb genes which build the R-body, a large protein structure which is crucial to deliver the toxin into the target cell’s cytoplasm. Thus, the outlier position of RebA, RebB, and RebD genes suggests a more recent HGT of these genes than the acquisition of the complete plasmid pKAP51 by *Caedibacter*, which is maybe even more evident when looking at the overall profile of plasmid- versus chromosomal encoded genes (Fig. 5C). The plasmid carries several transposons and transposases as well as phage-derived genes. It indeed has been speculated that it might derive from a phage genome^26^. The finding that HGT contributed to the evolution of the killer trait in *Paramecium* enforces our understanding of genetic transfer between species to rapidly confer new characteristics as in the system aphid-*Hamiltonella defensa*-phage APSE-2^29^. Another example involves the killer-effect in yeast strains which is based on the presence of two distinct mycoviruses which allow the secretion of toxic proteins to kill uninfected yeast cells as well as providing protective immunity^30^. Thus, in all these systems viruses confer the ability to kill predators or competitors.

However, the Reb encoded R-body only delivers the toxin to sensitive cells. Searching for the toxin itself, candidates are the different toxin-antitoxin systems (Fig. 5B) present in the *C. taeniospiralis* genome and even as part of the incomplete prophage (Fig. 4) as they could provide both toxicity and non-permanent immunity. Taking advantage of heterologous expression of R-bodies in *E. coli*^9^, potential toxin candidates can now be easily screened by co-expression in the same *E. coli* vector. As feeding of R-body producing bacteria does not cause lethal effects in paramecia^9^, the addition of the killer trait toxin should cause cell death in sensitive strains.

### The secretome is enriched in uncharacterized proteins

**Figure 6:**
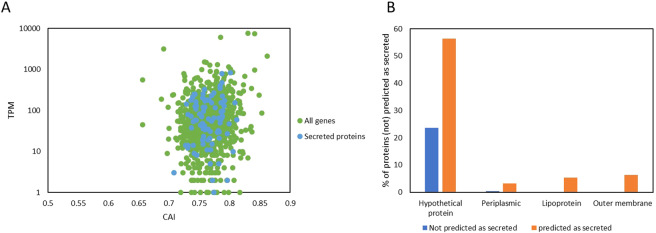
Predicted secretome of *Caedibacter taeniospiralis*. (**A**) Scatter plot of gene expression as transcripts per million (TPM) vs. codon adaptation (CAI). Protein coding genes that are predicted to be secreted by SignalIP are highlighted in light blue. (**B**) Ratio of potentially secreted proteins among different protein “classes” (hypothetical, periplasmic, lipoprotein and outer membrane protein) based on the amount of proteins that were predicted as secreted (total of 94 proteins) or not predicted as secreted (total of 993 proteins).

An alternative perspective on this endosymbiosis is the secretome analysis as all secreted proteins are in direct contact with the hosts cytoplasm and therefore the main candidates for intraspecific communication and adaptation. We performed *in silico* prediction of secreted proteins (Fig. 6A), many of them show high transcript levels (See Supplementary Table S2 for the full list of genes). From 95 proteins identified as secreted, 54 are hypothetical proteins and Fig. 6B indicates that the secretome shows clearly a lower annotation score meaning that reverse genetics can only describe potential functions for few secreted proteins. Among those is for example a component (IcmE) of a type IV secretion system. More parts (12 in total) of this large translocation machinery transporting proteins or protein/DNA complexes out of the symbionts’ cells are encoded on contigs CDBSP s01, s12, and s14. Type IV secretions systems are used by some pathogenic bacteria to inject virulence factors into their host cells^31^. However, the majority of secreted proteins are hypothetical, thus, their function remains unknown. This might simply be an indication for our limited knowledge of effector proteins in general, or instead due to a highly specialized secretome of this endosymbiont which likely constitutes not only effectors required for the alteration of the host’s transcriptome^10^ but also the antitoxin providing immunity against the killer trait. Thus, our data now allows to identify and to characterize these secreted proteins to gain deeper understanding into the molecular communication of the symbiosis and the identification of the killer trait toxin and resistance mechanism.

## Methods

### DNA isolation, library preparation and sequencing

*Paramecium tetraurelia* strain 51K (CCAP 1660/3F) infected with cytoplasmic *C. taeniospiralis* as verified by fluorescence *in situ* hybridisation with the species-specific probe Ctaen998^8^ was grown on beta-lactam hypersensitive *E. coli* Delta tolC^32^ to limit the contamination with food bacteria. Total DNA was isolated after ampicillin treatment^11^ and used for Illumina and ONT sequencing. Tagmentation^33^ was performed for Illumina library generation with different ratios of DNA/transposase (Supplementary Fig. S1) to optimize insert length in addition to two different polymerases (Q5, NEB and KAPA HiFi, Roche). After gel purification, Illumina MiSeq sequencing was carried out with a read-lenght of 2 × 300 nt. Reads were trimmed for adapters and low quality bases by the cutadapt (v1.4.1) wrapper trim galore (v0.3.3)^34^. Additionally, MinION library preparation and sequencing was performed by Seq-IT (Kaiserslautern, Germany) using a 1D2 Sequencing Kit (LSK-308, ONT) and a MKI vR9 MinION flow cell (FLO-MIN107, ONT) to obtain longer DNA reads in order to improve the genome assembly. In total, 1.099 Gbp of raw ONT data with a mean read length of 11955 bp were obtained.

### RNA isolation, library preparation and sequencing

Total RNA was isolated from cultures after antibiotics treatment using Tri-Reagent (Sigma). After additional DNAse digestion, RNA samples were depleted for eukaryotic and bacterial rRNAs by subsequent usage of the Yeast Ribo-Kit and the gram-negative bacterial Ribo-Zero Kits (Illumina). After additional depletion of poly(A) RNAs, directional RNA libraries were generated from the left-over RNA using the NEB ultra directional RNA library prep Kit (NEB). Libraries were sequenced on a Illumina HiSeq2500 Platform and trimmed as described above. RNA reads have been deposited under ENA accession number PRJEB36201.

### Genome assembly and gene annotation

Genome assembly and gene annotation was carried out as described^11^ using Illumina reads. Additionally, ONT reads were assembled using the Canu assembler^35^ version 1.7 with default parameters. The resulting assembly was compared to and used for joining the contigs derived from the Illumina-only assembly in Geneious version 11.1.2^36^. reducing the number of contigs from 24 to 18.

Gene annotation improvement and operon definition was carried out using Rockhopper v2.03^15^. To prevent inclusion of reads derived from the host, dual RNA-Seq reads were first mapped to the *Paramecium* genome using Bowtie2^37^. Subsequently, mapping reads were subtracted. The remaining reads were then analyzed by Rockhopper with default settings. Genes that were annotated neither during the first annotation nor by the Rockhopper tool, but showed a distinct mRNA coverage signal, were annotated manually and verified via BLAST search of the corresponding nucleotide and protein sequences.

This Whole Genome Shotgun project has been deposited at DDBJ/ENA/GenBank under the accession PGGB00000000. The version described in this paper is version PGGB02000000. We also published the corresponding Geneious format including all annotations as well as the .fasta- and .gff-files of the genome at Zenodo (https://zenodo.org/; 10.5281/zenodo.3372731).

Transcripts per million (TPM) values of the *Caedibacter* genes were calculated by Rockhopper using the same RNA-Seq reads mentioned above.

### Phylogenetic analyses and operon synteny

Phylogenetic distances were inferred based on either 16S rRNA gene sequence respectively a selection of 19 conserved bacterial single copy genes. The 16S rRNA gene sequence of *C. taeniospiralis* was aligned against its closest relatives with sequenced genomes (Supplementary Table S1). Phylogenetic trees were calculated using Bayesian Inference (BI; MrBayes 3.2.6^38^; with a burn-in of 25% after 1,000,000 generations, and Maximum Likelihood (ML) with 1,000 bootstrap pseudoreplicates (PHYML 2.4.5^39^). Furthermore, 19 conserved bacterial single copy genes were identified and extracted from each genome using AmphoraNet^40^. Protein sequences (Supplementary Table S3) were concatenated and aligned (MUSCLE version 3.8.31^41^) comprising 6072 characters. BI phylogenetic analysis of AMPHORA concatenated multiprotein sequences were carried as described above. For pairwise genome comparisons, the average nucleotide identity was calculated using the BLAST+-based approach (ANIb) by JSpeciesWS version 3.1.2^42^. A heatmap was generated using CIMminer (http://discover.nci.nih.gov/cimminer). Digital DNA-DNA hybridization (dDDH) values were estimated with the Genome-to-Genome Distance Calculator (using GGDC 2.1 BLAST+^43^) applying formula 2 which accounts for incomplete genome sequences. Genomes used for the *in silico* analyses are listed in Supplementary Table S1. Synteny analysis of the genetic context of the *str*-operon between the different indicated organisms was carried out using the Mauve genome alignment algorithm^44^ running on default settings.

### Genomic and post-genomic analyses

Prophage and CRISPR identification: Prophages were searched for using the PHASTER web server (from: http://phaster.ca/)^28^. Clustered Regularly Interspaced Short Palindromic Repeats (CRISPR) were identified by CRISPRCasFinder (from: https://crisprcas.i2bc.pariss)^45^.

CAI prediction/estimation: A codon usage table of *Caedibacter* was created by a web-based codon usage calculator^46^. The codon adaptation index (CAI) of *Caedibacter* genes was calculated using the web-based CAI calculator from EMBOSS (http://www.bioinformatics.nl/cgibin/emboss/help/cai).

Secretome analysis: Prediction of potentially secreted proteins was performed by submitting a fasta-file containing the amino-acid sequence from all protein-coding genes of the *Caedibacter* genome to the SignalIP v5.0 webtool^47^ using the default thresholds for signal peptide prediction.

## Supplementary information

Supplementary Information.
