# Supplementary material for: Dual-Seq reveals genome and transcriptome of Caedibacter taeniospiralis, obligate endosymbiont of Paramecium
Source: Sci Rep. 2020 Jun 16;10:9727. doi: 10.1038/s41598-020-65894-1 (PMC7297999; doi:10.1038/s41598-020-65894-1)
Supplement: Supplementary file 1 — Supplementary Information. [file 41598_2020_65894_MOESM1_ESM.pdf]

Dual-Seq reveals the genome and transcriptome of *Caedibacter taeniospiralis*, obligate endosymbiont of *Paramecium*

by

Marcello Pirritano, Nestor Zaburannyi, Katrin Grosser, Gilles Gasparoni, Rolf Müller,

Martin Simon, Martina Schrallhammer

Supplementary Figures and Tables

Supplementary Figure S1

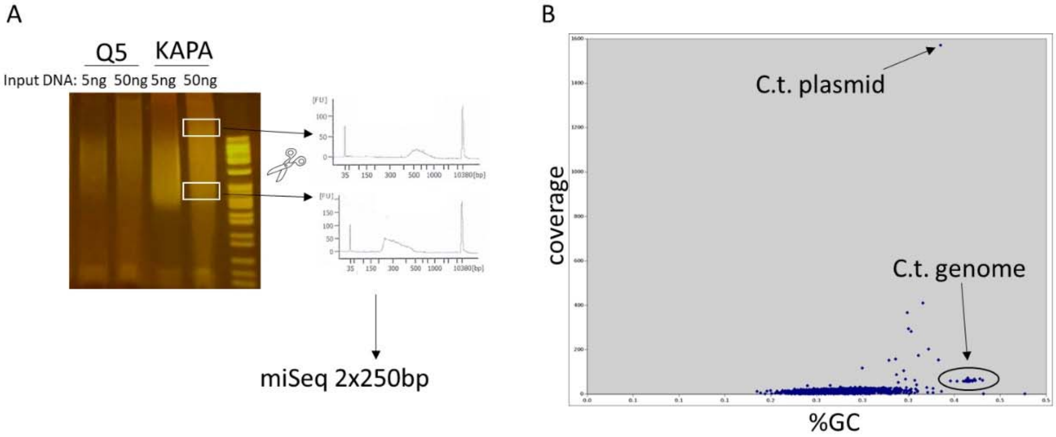

Supplementary Fig. S1: (A) Library preparation strategy: Different amounts of input DNA were used for Tagmentation. We used the Q5 (NEB) and KAPA (Roche) DNA polymerases for library amplification and extracted fragments from PAGE. Long fragments of ~500bp ~were pair end sequenced on an Illumina MiSeq platform. (B) Scatter plot of assembled scaffolds, coverage vs. GC percentage. Host chromosomes cluster at low coverage at 20-30% GC. *Caedibacter* DNA shows higher coverage, due to the high infection rate and a higher GC percentage. The *Caedibacter* plasmid shows higher coverage of ~1600x.

Supplementary Figure S2

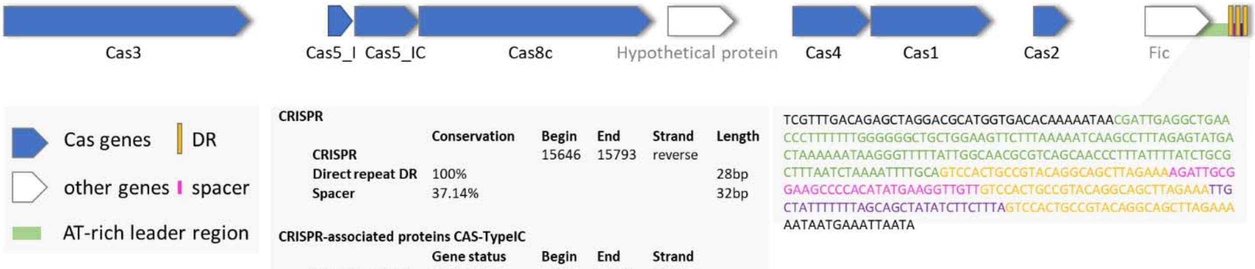

CRISPR

	Conservation	Begin	End	Strand	Length
**CRISPR**		15646	15793	reverse	
**Direct repeat DR**	100%				28bp
**Spacer**	37.14%				32bp

CRISPR-associated proteins CAS-TypeIC

	Gene status	Begin	End	Strand
**Cas2_0_I-II-III-V**	accessory	17081	17374	reverse
**Cas1_0_IC**	mandatory	17714	18703	reverse
**Cas4_0_I-II**	accessory	18700	19335	reverse
**Cas8c_0_IC**	mandatory	20476	22365	reverse
**Cas5_0_IC**	mandatory	22362	22904	reverse
**Cas5_0_I**	accessory	22926	23108	reverse
**Cas3_0_I**	accessory	23732	25747	reverse

TCGTTTGACAGAGCTAGGACGCATGGTGACACAAAAATAACGATTGAGGCTGAA
CCCTTTTTTTGGGGGGCTGCTGGAAGTTCTTTAAAAATCAAGCCTTTAGAGTATGA
CTAAAAAATAAGGGTTTTTATTGGCAACGCGTCAGCAACCCTTTATTTTATCTGCG
CTTTAATCTAAAATTTTGCAGTCCACTGCCGTACAGGCAGCTTAGAAAAGATTGCG
GAAGCCCCACATATGAAGGTTGTTGTCCACTGCCGTACAGGCAGCTTAGAAATTG
CTATTTTTTTAGCAGCTATATCTTCTTTAGTCCACTGCCGTACAGGCAGCTTAGAAA
AATAATGAAATTAATA

Supplementary Fig. S2: Scheme of the CRISPR locus (localized on CDBSP_s01) comprising seven *cas* genes, an AT-rich leader sequence (146bp, in green) and three direct repeat sequences (28bp, in orange) separated by unique sequences (spacers, 32bp, in pink and purple).

Supplementary Table S1

Supplementary Table S1: Phylogenetic analysis of *Caedibacter taenospiralus* using the ANIb and dDDH method.

ANIb

			**1**	**2**	**3**	**4**	**5**	**6**
**1**	*Caedibacter taeniospiralis*	PGGB02000000	**100**					
**2**	*Fangia hongkongensis*	NZ_KB902239.1	69.38	**100**				
**3**	*Cysteiniphilum litorale*	QLIQ01000001-QLIQ01000198	74.70	70.37	**100**			
**4**	*Fastidiosibacter lacustris*	QLIR01000001-QLIR01000062	73.56	69.96	73.71	**100**		
**5**	*Facilibium subflavum*	QLIT01000001-QLIT01000389	70.08	70.14	70.56	70.73	**100**	
**6**	*Cysteiniphilum halobium*	QLIU01000001-QLIU01000159	74.71	69.96	84.77	73.59	70.66	**100**
	ANIb values above the 95% cut-off for species delineation are shown in bold type.							

dDDH

			**1**	**2**	**3**	**4**	**5**	**6**
**1**	*Caedibacter taeniospiralis*	PGGB02000000	**100**					
**2**	*Fangia hongkongensis*	NZ_KB902239.1	23.3	**100**				
**3**	*Cysteiniphilum litorale*	QLIQ01000001-QLIQ01000198	21.9	22.3	**100**			
**4**	*Fastidiosibacter lacustris*	QLIR01000001-QLIR01000062	21.7	22.3	21.7	**100**		
**5**	*Facilibium subflavum*	QLIT01000001-QLIT01000389	21.5	21.4	22.0	22.8	**100**	
**6**	*Cysteiniphilum halobium*	QLIU01000001-QLIU01000159	22.2	20.7	29.1	21.0	23.7	**100**
	dDDH values above the 70% cut-off for species delineation are shown in bold type.							

Supplementary Table S2

Supplementary Table S2: List of genes that were predicted to be secreted using the SignalIP-5.0 webtool. Additionally, CAI and TPM are indicated for each gene.

**Gene**	**CAI**	**TPM**
CDBSP_s01_-_DNA_pantothenate_metabolism_flavoprotein_CDS	0.758	43
CDBSP_s01_-_Hypothetical_protein_CDS_12	0.78	29
CDBSP_s01_-_Membrane-bound_lytic_murein_transglycosylase_CDS	0.731	14
CDBSP_s01_-_NAD(P)H-dependent_glycerol-3-phosphate_dehydrogenase_CDS	0.727	14
CDBSP_s01_-_Outer-membrane_lipoprotein_carrier_protein_CDS	0.75	83
CDBSP_s02_-_Hypothetical_protein_CDS_10	0.74	14
CDBSP_s02_-_Hypothetical_protein_CDS_11	0.752	0
CDBSP_s02_-_Hypothetical_protein_CDS_12	0.782	0
CDBSP_s02_-_Hypothetical_protein_CDS_14	0.786	61
CDBSP_s02_-_Hypothetical_protein_CDS_29	0.796	27
CDBSP_s02_-_Hypothetical_protein_CDS_30	0.744	162
CDBSP_s02_-_Hypothetical_protein_CDS_7	0.782	66
CDBSP_s02_-_Peptidase_S8_and_S53_subtilisin_kexin_sedolisin_CDS	0.782	5
CDBSP_s02_-_Peptidoglycan_glycosyltransferase_CDS	0.783	21
CDBSP_s02_-_Soluble_lytic_murein_transglycosylase_CDS	0.786	93
CDBSP_s02_-_Uncharacterized_ABC_transporter__auxiliary_component_CDS	0.756	119
CDBSP_s03_-_HesA_MoeB_ThiF_family_protein_CDS	0.783	30
CDBSP_s03_-_Hypothetical_protein_CDS_16	0.759	121
CDBSP_s03_-_Hypothetical_protein_CDS_17	0.791	130
CDBSP_s03_-_Hypothetical_protein_CDS_19	0.769	11
CDBSP_s03_-_Hypothetical_protein_CDS_20	0.743	125
CDBSP_s03_-_Hypothetical_protein_CDS_22	0.739	215
CDBSP_s03_-_OmpA_family_protein_CDS	0.781	32
CDBSP_s03_-_Peptide-methionine_(S)-S-oxide_reductase_CDS	0.727	0
CDBSP_s04_-_Hypothetical_protein_CDS_10	0.758	328
CDBSP_s04_-_Hypothetical_protein_CDS_15	0.768	5
CDBSP_s04_-_Hypothetical_protein_CDS_21	0.816	0
CDBSP_s04_-_Hypothetical_protein_CDS_35	0.736	72
CDBSP_s04_-_Hypothetical_protein_CDS_36	0.771	15
CDBSP_s04_-_Hypothetical_protein_CDS_39	0.787	482
CDBSP_s04_-_Hypothetical_protein_CDS_4	0.736	178
CDBSP_s04_-_Hypothetical_protein_CDS_41	0.756	15
CDBSP_s04_-_Hypothetical_protein_CDS_5	0.764	35
CDBSP_s04_-_Hypothetical_protein_CDS_6	0.786	152
CDBSP_s04_-_Hypothetical_protein_CDS_7	0.812	59
CDBSP_s04_-_Hypothetical_protein_CDS_9	0.743	249
CDBSP_s04_-_IcmE_protein_CDS	0.796	2
CDBSP_s04_-_Periplasmic_binding_protein_CDS	0.765	62
CDBSP_s04_-_periplasmic_or_secreted_lipoprotein_CDS	0.77	232
CDBSP_s05_-_Citrate_transporter_CDS	0.708	3
CDBSP_s05_-_Hypothetical_protein_CDS_10	0.746	8

CDBSP_s05_-_Hypothetical_protein_CDS_3	0.788	70
CDBSP_s05_-_Hypothetical_protein_CDS_4	0.768	31
CDBSP_s05_-_Hypothetical_protein_CDS_5	0.756	252
CDBSP_s05_-_Hypothetical_protein_CDS_9	0.731	12
CDBSP_s05_-_lipoprotein_CDS	0.774	270
CDBSP_s05_-_Outer_membrane_protein_assembly_factor_CDS	0.761	177
CDBSP_s05_-_Outer_membrane_protein_assembly_factor_CDS	0.761	177
CDBSP_s05_-_Outer_membrane_protein_CDS	0.809	154
CDBSP_s06_-_Beta-lactamase_CDS	0.764	19
CDBSP_s06_-_Hypothetical_protein_CDS_16	0.587	1679
CDBSP_s06_-_Hypothetical_protein_CDS_4	0.741	246
CDBSP_s06_-_Peptidyl-prolyl_cis-trans_isomerase_CDS	0.787	795
CDBSP_s06_-_transmembrane_protein_CDS	0.737	38
CDBSP_s06_-_Zinc_ABC_transporter__periplasmic-binding_protein_CDS	0.77	55
CDBSP_s07_-_Hypothetical_protein_CDS_10	0.757	23
CDBSP_s07_-_Hypothetical_protein_CDS_13	0.766	0
CDBSP_s07_-_Hypothetical_protein_CDS_14	0.755	203
CDBSP_s07_-_Hypothetical_protein_CDS_7	0.772	2
CDBSP_s07_-_Hypothetical_protein_CDS_9	0.756	36
CDBSP_s07_-_Lipoprotein_VacJ-like_protein_CDS	0.789	99
CDBSP_s07_-_OmpA_MotB_domain_protein_CDS	0.801	827
CDBSP_s07_-_Protein_tyrosine_phosphatase_CDS	0.799	47
CDBSP_s08_-_18K_peptidoglycan-associated_outer_membrane_lipoprotein_CDS	0.788	222
CDBSP_s08_-_Hypothetical_protein_CDS_10	0.745	58
CDBSP_s08_-_Hypothetical_protein_CDS_13	0.745	128
CDBSP_s08_-_Hypothetical_protein_CDS_3	0.763	11
CDBSP_s08_-_Hypothetical_protein_CDS_4	0.783	0
CDBSP_s08_-_Import_inner_membrane_translocase_subunit_Tim44_CDS	0.784	361
CDBSP_s08_-_Outer-membrane_lipoprotein_CDS	0.743	57
CDBSP_s08_-_Protein_CDS	0.757	95
CDBSP_s09_-_DNA_RNA_endonuclease_G_CDS	0.741	9
CDBSP_s09_-_Hypothetical_protein_CDS_22	0.784	401
CDBSP_s09_-_Phosphatidylcholine-hydrolyzing_phospholipase_C_CDS	0.774	1
CDBSP_s09_-_Surface_antigen_(D15)_CDS	0.755	19
CDBSP_s11_-_Hypothetical_protein_CDS	0.759	170
CDBSP_s11_-_oppA_CDS	0.767	21
CDBSP_s12_-_Hypothetical_protein_CDS	0.776	117
CDBSP_s12_-_Hypothetical_protein_CDS_2	0.784	50
CDBSP_s12_-_Hypothetical_protein_CDS_9	0.754	71
CDBSP_s12_-_Serine-type_D-Ala-D-Ala_carboxypeptidase_CDS	0.754	46
CDBSP_s13_-_Hypothetical_protein_CDS_2	0.77	152
CDBSP_s13_-_Hypothetical_protein_CDS_4	0.755	48
CDBSP_s13_-_Hypothetical_protein_CDS_8	0.729	143
CDBSP_s13_-_Peptidase_M23_CDS	0.774	65
CDBSP_s14_-_Hypothetical_protein_CDS_2	0.759	34
CDBSP_s14_-_Lin2838_protein_CDS	0.784	341

CDBSP_s14_-_Predicted_UDP-glucose_6-dehydrogenase_CDS	0.752	44
CDBSP_s14_-_Protoporphyrinogen_IX_oxidase__aerobic_CDS	0.74	54
CDBSP_s15_-_Outer_membrane_protein_tolC_CDS	0.792	174
CDBSP_s15_-_TPR_repeat_protein_CDS	0.744	108
CDBSP_s17_-_Hypothetical_protein_CDS	0.731	80
CDBSP_s17_-_Hypothetical_protein_CDS_2	0.806	10
CDBSP_s18_-_Hypothetical_protein_CDS_12	0.766	180
CDBSP_s18_-_Hypothetical_protein_CDS_4	0.788	72

Supplementary Table S3

Supplementary Table S3: List of marker genes that has been used for phylogenetic analysis.

	Caedibacter taeniospiralis	*Allofrancisella guangzhouensis*	*Fangia hongkongensis*	*Francisella halioticida*	*Francisella hispaniensis*
Genome accession	PGGB02000000	CP010427.1	NZ_KB902239.1	NZ_CP022132.1	CP018093.1
Marker gene					
dnaG	CDBSP_8980	CP010427.1_4120	ARAW01000006.1_2359	NZ_CP022132.1_14974	CP018093.1_11902
frr	CDBSP_6510	CP010427.1_2356	ARAW01000001.1_46	NZ_CP022132.1_1888	CP018093.1_1052
infC	CDBSP_10410	CP010427.1_3888	ARAW01000007.1_2110	NZ_CP022132.1_3189	CP018093.1_3667
nusA	CDBSP_02880	CP010427.1_14299	ARAW01000019.1_604	NZ_CP022132.1_11426	CP018093.1_9628
pgk	CDBSP_3640	CP010427.1_5650	ARAW01000006.1_2342	NZ_CP022132.1_4550	CP018093.1_2897
pyrG	CDBSP_7090	CP010427.1_2437	ARAW01000002.1_3282	NZ_CP022132.1_2069	CP018093.1_1155
rplA	CDBSP_8160	CP010427.1_2514	ARAW01000003.1_2547	NZ_CP022132.1_1065	CP018093.1_10063
rplK	CDBSP_8170	CP010427.1_2512	ARAW01000003.1_2544	NZ_CP022132.1_1063	CP018093.1_10062
rplL	CDBSP_8140	CP010427.1_2520	ARAW01000003.1_2552	NZ_CP022132.1_1070	CP018093.1_10067
rplM	CDBSP_8530	CP010427.1_10824	ARAW01000002.1_446	NZ_CP022132.1_17736	CP018093.1_14280
rplS	CDBSP_5660	CP010427.1_2561	ARAW01000007.1_1843	NZ_CP022132.1_19347	CP018093.1_10118
rplT	CDBSP_10390	CP010427.1_3891	ARAW01000007.1_2113	NZ_CP022132.1_3194	CP018093.1_3670
rpmA	CDBSP_10600	CP010427.1_8831	ARAW01000010.1_985	NZ_CP022132.1_2922	CP018093.1_11081
rpoB	CDBSP_8120	CP010427.1_2530	ARAW01000003.1_2566	NZ_CP022132.1_1078	CP018093.1_10072, CP018093.1_10079
rpsB	CDBSP_6480	CP010427.1_2349	ARAW01000001.1_36	NZ_CP022132.1_1882	CP018093.1_1047
rpsI	CDBSP_8540	CP010427.1_10825	ARAW01000002.1_449	NZ_CP022132.1_17738	CP018093.1_14281
rpsJ	CDBSP_0890	CP010427.1_2386	ARAW01000014.1_220	NZ_CP022132.1_1913	CP018093.1_1076
smpB	CDBSP_9460	CP010427.1_9875	ARAW01000021.1_505	NZ_CP022132.1_16840	CP018093.1_13483
tsf	CDBSP_6490	CP010427.1_2351	ARAW01000001.1_41	NZ_CP022132.1_1884	CP018093.1_1050

	Francisella noatunensis subsp. *noatunensis*	*Francisella persica*	*Francisella philomiragia* subsp. *philomiragia*	*Francisella tularensis* subsp. *tularensis*
Genome accession	NZ_LTDO00000000.1	CP013022.1	CP000937.1	AJ749949.2
Marker gene				
dnaG	LTDO01000007.1_267	CP013022.1_8877	CP000937.1_8526	AJ749949.2_12349
frr	LTDO01000002.1_1298	CP013022.1_13829	CP000937.1_16963	AJ749949.2_1373
infC	LTDO01000006.1_1372	CP013022.1_15118	CP000937.1_19236	AJ749949.2_3380
nusA	LTDO01000003.1_3012	CP013022.1_2933	CP000937.1_5267	AJ749949.2_201
pgk	LTDO01000003.1_4621	CP013022.1_4202	CP000937.1_7057	AJ749949.2_10800
pyrG	LTDO01000002.1_1448	CP013022.1_13926	CP000937.1_17112	AJ749949.2_15788
rplA	LTDO01000003.1_3380	CP013022.1_3256	CP000937.1_5666	AJ749949.2_567
rplK	LTDO01000003.1_3379	CP013022.1_3255	CP000937.1_5665	AJ749949.2_565
rplL	LTDO01000003.1_3385	CP013022.1_3261	CP000937.1_5672	AJ749949.2_572
rplM	LTDO01000001.1_386	CP013022.1_443	CP000937.1_12116	AJ749949.2_5649
rplS	LTDO01000003.1_3432	CP013022.1_3297	CP000937.1_5715	AJ749949.2_622
rplT	LTDO01000006.1_1376	CP013022.1_15120	CP000937.1_19240	AJ749949.2_3384
rpmA	LTDO01000010.1_1065	CP013022.1_8363	CP000937.1_19069	AJ749949.2_3233
rpoB	LTDO01000003.1_3396	CP013022.1_3273	CP000937.1_5684	AJ749949.2_587
rpsB	LTDO01000002.1_1293	CP013022.1_13823	CP000937.1_16957	AJ749949.2_1368
rpsI	LTDO01000001.1_387	CP013022.1_444	CP000937.1_12117	AJ749949.2_5650
rpsJ	LTDO01000002.1_1323	CP013022.1_13858	CP000937.1_16995	AJ749949.2_1398
smpB	LTDO01000006.1_456	CP013022.1_7264	CP000937.1_271	AJ749949.2_5239
tsf	LTDO01000002.1_1295	CP013022.1_13826	CP000937.1_16959	AJ749949.2_1370

	Fastidiosibacter lacustris	*Facilibium subflavum*	*Cysteiniphilum halobium*	*Cysteiniphilum litorale*
Genome accession	QLIR01000001-QLIR01000062	QLIT01000001-QLIT01000389	QLIU01000001-QLIU01000159	QLIQ01000001-QLIQ01000198
Marker gene				
dnaG	QLIR01000008.1_644	QLIT01000191.1_168	QLIU01000015.1_1149	QLIQ01000016.1_443
frr	QLIR01000003.1_760	QLIT01000100.1_136	QLIU01000055.1_89	QLIQ01000003.1_1107
infC	QLIR01000002.1_863	QLIT01000102.1_577	QLIU01000025.1_428	QLIQ01000024.1_12
nusA	QLIR01000004.1_410	QLIT01000013.1_308	QLIU01000070.1_55	QLIQ01000014.1_162
pgk	QLIR01000009.1_718	QLIT01000050.1_120	QLIU01000009.1_38	QLIQ01000027.1_87
pyrG	QLIR01000011.1_179	QLIT01000011.1_364	QLIU01000035.1_119	QLIQ01000009.1_953
rplA	QLIR01000004.1_153	QLIT01000137.1_56	QLIU01000036.1_389	QLIQ01000030.1_113
rplK	QLIR01000004.1_150	QLIT01000137.1_54	QLIU01000036.1_386	QLIQ01000030.1_109
rplL	QLIR01000004.1_158	QLIT01000137.1_61	QLIU01000036.1_393	QLIQ01000030.1_118
rplM	QLIR01000028.1_181	QLIT01000004.1_7	QLIU01000049.1_7	QLIQ01000002.1_228
rplS	QLIR01000002.1_953	QLIT01000276.1_1	QLIU01000012.1_270	QLIQ01000001.1_1789
rplT	QLIR01000002.1_867	QLIT01000102.1_582	QLIU01000025.1_431	QLIQ01000024.1_15
rpmA	QLIR01000003.1_733	QLIT01000191.1_120	QLIU01000071.1_247	QLIQ01000022.1_261
rpoB	QLIR01000004.1_171	QLIT01000137.1_81	QLIU01000036.1_408	QLIQ01000030.1_131
rpsB	QLIR01000003.1_751	QLIT01000100.1_125	QLIU01000055.1_83	QLIQ01000003.1_1098
rpsI	QLIR01000028.1_182	QLIT01000004.1_8	QLIU01000049.1_9	QLIQ01000002.1_229
rpsJ	QLIR01000007.1_248	QLIT01000115.1_436	QLIU01000005.1_65	QLIQ01000014.1_60
smpB	QLIR01000005.1_1005	QLIT01000099.1_336	QLIU01000023.1_383	QLIQ01000013.1_457
tsf	QLIR01000003.1_754	QLIT01000100.1_132	QLIU01000055.1_87	QLIQ01000003.1_1105
